# The *Passiflora tripartita* (Banana Passion) Fruit: A Source of Bioactive Flavonoid *C*-Glycosides Isolated by HSCCC and Characterized by HPLC–DAD–ESI/MS/MS

**DOI:** 10.3390/molecules18021672

**Published:** 2013-01-28

**Authors:** Mario J. Simirgiotis, Guillermo Schmeda-Hirschmann, Jorge Bórquez, Edward J. Kennelly

**Affiliations:** 1 Laboratorio de Productos Naturales, Departamento de Química, Universidad de Antofagasta, Casilla 170, Antofagasta, Chile; E-Mail: jborquez@uantof.cl; 2 Laboratorio de Química de Productos Naturales, Instituto de Química de Recursos Naturales, Universidad de Talca, Casilla 747, Talca, Chile; E-Mail: schmeda@utalca.cl; 3 Department of Biological Sciences, Lehman College and The Graduate Center, the City University of New York, 250 Bedford Park Boulevard West, Bronx, NY 10468, USA; E-Mail: edward.kennelly@lehman.cuny.edu

**Keywords:** banana passion fruits, *Passiflora tripartita*, *Passiflora tripartita* var. mollissima, tumbo, HPLC-MS, *C*-glycosyl flavonoids, phenolic compounds, antioxidants, High-Speed Countercurrent Chromatography, HSCCC

## Abstract

The banana passion fruit (*Passiflora tripartita* Breiter, Passifloraceae) known as “tumbo” is very appreciated in tropical and subtropical countries of South America. Methanolic extracts from peel and the fruit juice of *P. tripartita* growing in Chile were analyzed for antioxidant capacity as well as for flavonoid and phenolic content. A chromatographic method was developed for the rapid identification of the main phenolics in the samples by HPLC-DAD and HPLC-MS. The fast fingerprint analysis allowed the detection of eighteen flavonoid *C*-glycosides and four flavonoid *O*-glycoside derivatives which were characterized by UV spectra and ESI-MS-MS analysis. Several of the *C*-glycosides detected are structurally related to the orientin derivative 4′-methoxy-luteolin-8-*C*-(6″acetyl)-β-D-glucopyranoside (**31**), fully elucidated by spectroscopic methods. The antioxidant derivative **31** along with schaftoside, vicenin II, orientin and vitexin were isolated from the fruit extract by high-speed countercurrent chromatography (HSCCC). A suitable method for the preparative isolation of flavonol *C*-glycosides from “tumbo” extracts by HSCCC is reported. The pulp of the fruits showed good antioxidant capacity (12.89 ± 0.02 μg/mL in the DPPH assay). The peel presented the highest content of flavonoids (56.03 ± 4.34 mg quercetin/100 g dry weight) which is related to the highest antioxidant power (10.41 ± 0.01 μg/mL in the DPPH assay).

## 1. Introduction

The genus *Passiflora* from the Passifloraceae plant family comprises around 450 species originated from temperate and tropical South America. The best known edible *Passiflora* fruits are *Passiflora edulis* f. *edulis* and *P. edulis* f. *flavicarpa*, the purple and yellow passion fruits. The passion flower genus or passion vine is also known to produce cyanogenic glycosides [1]. The leaves of several Passifloraceae are used worldwide in traditional medicine or in phytotherapy as anti-inflammatory [2] anxiolitic and sedative substances [3]. The biological activity was attributed, at least in part, to their content in *C*-glycosyl flavonoids [4]. Some worldwide consumed edible fruits such as lemon [5] and lime (*Citrus aurantifolia*) did not contain detectable amounts of *C*-glycosides [6] but they occur in medicinal plants used for similar indications than *Passiflora*, including hawthorn (*Crataegus monogyna* Jacq. (Lindt.) [7,8] and lemongrass (*Cymbopogon citratus*) [9]. 

The banana passion fruit, known in northern Chile as “tumbo” (*Passiflora tripartita* var. mollissima (Kunth) Holm-Niels. & P. Jørg., Passifloraceae), is a species native from the Andes. It grows from Venezuela to Bolivia at 1,800–3,600 m above the sea level in tropical high forests and has been naturalized in Chile, Mexico, New Zealand, Australia and the United States of America. The aromatic fruits, consumed from prehispanic times, are very appreciated for the pleasant taste and acidic fruit juice. 

Previous studies on the fruits revealed the presence of the cyanogenic glycoside prunasin (0.7 mg/Kg) [10], eugenyl *β*-D-glucoside [11], volatile hydrocarbons including mono and sesquiterpenes, ketones, aldehydes and esters in the fruits [12] which were identified by HRGC and HRGC-MS. Recently, the ascorbic acid content of this fruit was reported, and the fruit was considered a rich source of vitamin C (40 mg per 100 g of edible fruit) [13]. The aerial parts ethanolic extract showed hypoglycaemic activity [14] and antibacterial effect [15]. The pulp of a fruit sample collected in Perú showed higher antioxidant capacity in the DPPH assay (41.18 μmol/g Trolox equivalents (TE) fresh weight) than the noni fruit (*Morinda citrifolia*, 3.48 μmol/g TE fresh weight) [16]. The fruits of *P. tripartita* still have a limited production in Chile, being cultivated mainly in the northern part of the country due to climate constraints. The investigation of the bioactive phenolics composition and antioxidant capacity of tumbo fruits will provide potential consumers better information on the nutraceuticals of this South American species and encourage production.

High-speed countercurrent chromatography (HSCCC) is an all-liquid chromatographic technique which does not use a solid support as the stationary phase, allowing the injected sample to be recovered completely, with no oxidation or loss of bioactive compounds due to adsorption in a solid stationary phase. In the present work and following previous studies on this technique [17,18] we report the isolation of five mayor compounds, the antioxidant activity and the qualitative-quantitative phenolic profile of the edible parts and peel of tumbo fruits cultivated in northern Chile. The detection and characterization of eighteen flavonoid *C*-glycosides and other phenolics from *P. tripartita *by a combination of NMR, UV and ESI-MS-MS analysis is described. Some of the structures detected are related to a rare orientin derivative isolated from the aerial parts of this plant and previously elucidated by spectroscopic methods (HR 1D and 2D NMR) [19].

## 2. Results and Discussion

**Figure 1:**
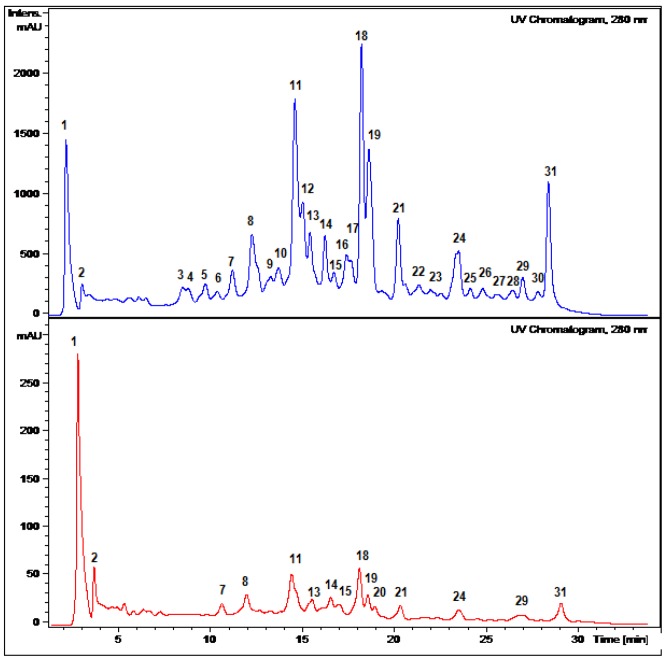
HPLC DAD chromatograms at 280 nm of *Passiflora tripartita* (“tumbo”) peel (upper) and juice-pulp (lower).

The MeOH extracts of pulp and juice (edible part) as well as peel from *P. tripartita *var. mollissima (tumbo) were evaluated for antioxidant power by the DPPH scavenging activity and the ferric reducing antioxidant power assay (FRAP). Both peel and edible parts showed high antioxidant power (Table 1). The compounds responsible for the antioxidant activity of the extracts were isolated by centrifugal countercurrent chromatography (HSCCC) and were identified by spectroscopic and spectrometric means. An HPLC DAD-MS analysis of the extracts was undertaken to provide baseline information for further studies on the chemical variability of the species and to serve as a fingerprint (Figure 1) for comparison with other *Passiflora* species.

**Table 1:** Antioxidant power as determined by the DPPH bleaching assay, ferric reducing antioxidant power (FRAP), total phenolic content (TPC), total flavonoid content (TFC) and yield of tumbo fruits extracts and isolated compounds.

DPPH ^a^	FRAP ^b^	TPC ^c^	TFC ^d^	Yield ^e^	Fruit part
10.41 ± 0.01	462.47 ± 0.49	56.03 ± 4.34	140.17 ± 4.23	5.24	Peel
12.89 ± 0.02	85.78 ± 0.12	22.57 ± 1.43	77.16 ± 8.4	4.07	Pulp and juice
					Compound
65.40 ± 0.24	393.97 ± 0.31	-	-	0.06	11
44.86 ± 0.13	438.23 ± 0.28	-	-	0.07	13
24.24 ± 0.11	543.72 ± 0.43	-	-	0.04	16
1.62 ± 0.01	637.84 ±0.65	-	-	0.13	19
3.69 ± 0.04	586.48 ± 0.18	-	-	0.14	31
1.16 ± 0.01	729.37 ± 0.48	-	-	-	^f^ Gallic acid

^a^ Expressed as IC_50_ in µg/mL. ^b^ Expressed as mmol Trolox/100 g dry weight. ^c^ Expressed as mg gallic acid/100 g dry weight. ^d^ Expressed as mg quercetin/100 g dry weight. ^e^ Yield expressed as % w/w dry weight. All values are mean ± S.D. (n = 3). ^f^ Used as standard antioxidant. All values in the same column are significantly different (*p* < 0.05).

### 2.1. Isolation of Phenolic Compounds

**Figure 2:**
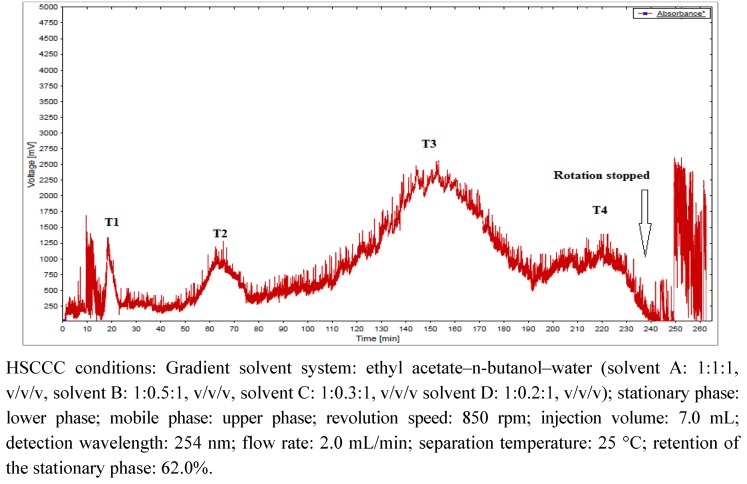
HSCCC chromatogram at 254 nm of crude tumbo (*Passiflora tripartita*) peel extract.

**Figure 3:**
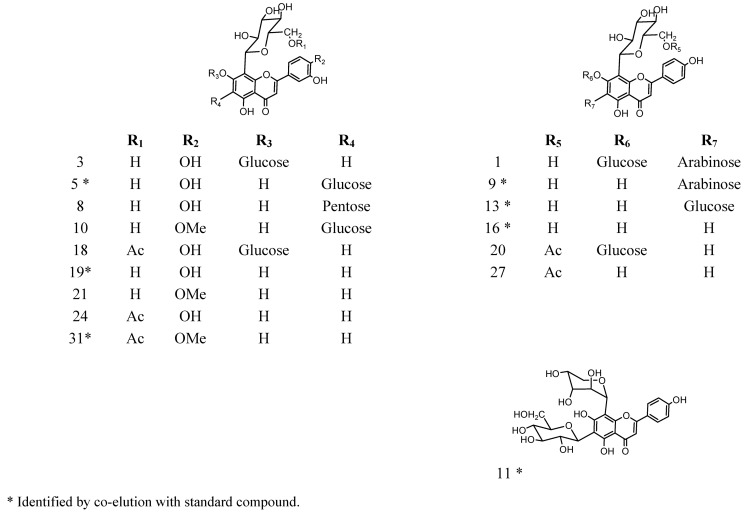
Proposed structures of luteolin and apigenin derivatives from *Passiflora tripartita* fruits identified by HPLC-DAD-ESI-MS.

The HSCCC chromatogram of the tumbo peel extract at 254 nm (Figure 2) shows four peaks (1–4) which were separately collected (see Experimental section) and purified by permeation on Sephadex LH-20 using MeOH as eluent. Five pure flavonoid *C*-glycosides were isolated (Figure 3) and identified by spectroscopic methods and HPLC–DAD-ESI-MS-MS analysis in comparison with authentic standards. 

Compound **11**: apigenin (8-*C*-α-L-arabinosyl) 6-*C*-*β*-D-glucopyranoside (schaftoside). The yield was 69.18 mg/kg fresh weight from the peel. Negative ESIMS: *m/z* 563 [M−H]^−^. The ^1^H-NMR and ^13^C-NMR data are consistent with published data [20]. 

Compound **13**: apigenin-6,8-di-*C*-*β*-D-glucopyranoside (vicenin II, 14.0 mg, Figure 3). The yield was 80.72 mg/kg fresh weight from the peel. Negative ESIMS: *m/z* 593 [M−H]^−^. The ^1^H-NMR (some glucoside signals overlapped) and ^13^C-NMR data are consistent with published data [21].

The identity of compounds **11** and **13** was confirmed by HPLC spiking experiments with an authentic sample (standard compound).

**Figure 4:**
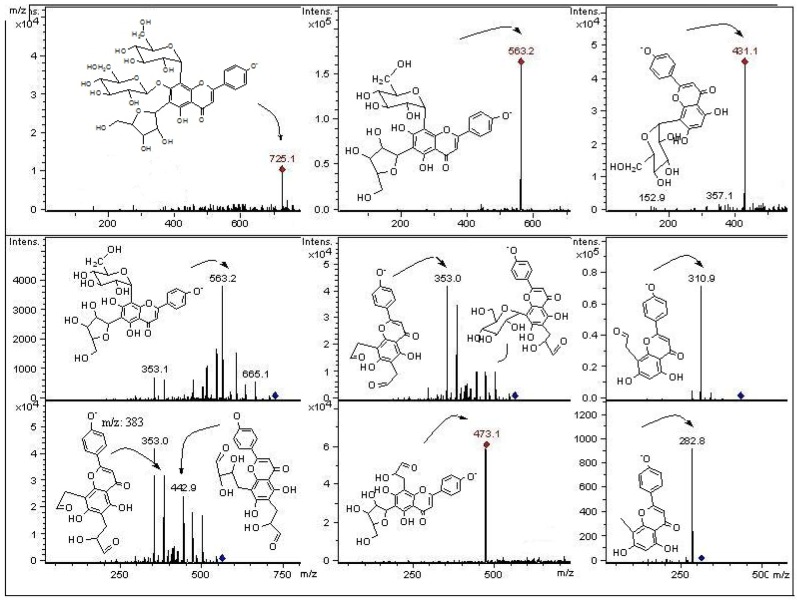
Structures, fragmentation, full ESI-MS and MS-MS spectra of peaks 1, 9, and 16.

Compound **16**: apigenin-8-*C*-*β*-D-glucopyranoside (vitexin, 7.0 mg, Table 1, Figure 3 and Figure 4). The yield was 40.36 mg/kg fresh weight from the peel. Negative ESIMS: *m/z* 431 [M−H]^−^. The ^1^H NMR and ^13^C-NMR data are in agreement with literature [22].

Compound **19**: luteolin-8-*C*-*β*-D-glucopyranoside (orientin, 24.0 mg, Figure 3). The yield was 138.32 mg/kg fresh weight from the peel. Negative ESIMS: *m/z* 447 [M−H]^−^. The ^1^H-NMR and ^13^C-NMR data agree with published data [23].

**Figure 5:**
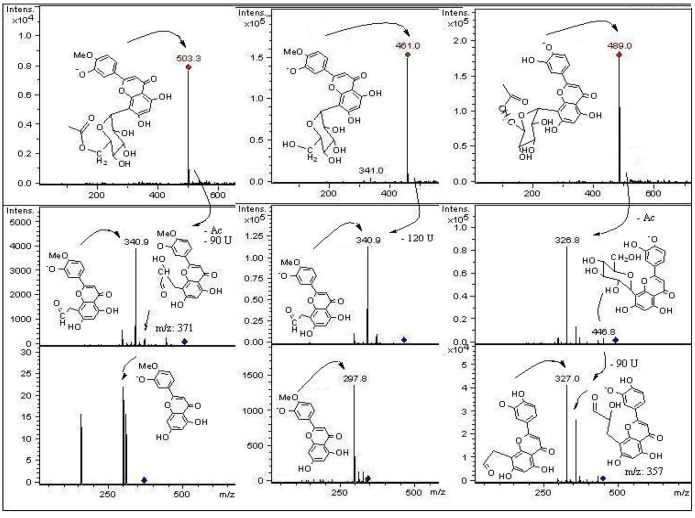
Structures, fragmentation, Full ESI-MS and MS-MS spectra of peaks 31, 21 and 24.

Compound **31**: 4′-methoxyluteolin-8-*C*-(6′′acetyl)-*β*-D-glucopyranoside (acetylmethylorientin, 27.0 mg, Figure 3 and Figure 5). The yield was 155.67 mg/kg fresh weight from the peel. Negative ESIMS: *m/z* 503 [M−H]^−^. The NMR data of this orientin derivative (^1^H, Figures S1–S4 and ^13^C) agree with literature [19,23].

### 2.2. HPLC-DAD and ESI Identification of Phenolic Compounds in Tumbo Fruits

The phenolic compounds occurring in *P. tripartita* fruits were investigated by high-performance liquid chromatography coupled with UV diode array detector (HPLC-DAD) and electrospray ionization with mass spectrometry detection (HPLC-ESI/MS). The extracts from fruit peel and pulp were dissolved in MeOH:water 7:3 (1 mg/mL) and 10 μL were injected into the HPLC system to obtain HPLC-DAD chromatograms (Figure 1). Several different gradients were employed but better separation between peaks **11**–**13** and **18**–**19** (Figure 1) could not be achieved under our experimental conditions. For mass spectrometry analysis and identification, all compounds were detected in both ESI positive and negative modes. However, the acidic nature (phenols) of the constituents besides higher percentage of ionization made easier the analysis in negative mode. In addition, the mobile phase employed had to be acidic in order to avoid the broadening of peaks due to the presence of both the neutral and deprotonated forms of the phenolic groups and also improve the retention of those compounds in the reverse phase C-18 column.

In mass spectrometry, *C*-glycosyl flavones experiment cross-ring cleavages of sugar residues yielding main signals (ions produced by losses of 60, 90 and 120 U) [9,24] that allowed differentiation with *O*-glycosyl flavones (losses of 162 U for hexose, 146 U for rhamnose and 132 U for pentose moieties, respectively) [25]. In this work we report glycosyl flavones with both characteristic signals, due to the presence of both *C*- and *O*- glycosydic linkages as previously reported to occur in some edible plants producing *C*-glycosyl flavonoids [9]. For the *C*-glycosides (Figure 3) ESI-MS data was in agreement with the proposed fragmentation [26]. The analysis showed that compounds **10**, **18**, **21** and **24** were structurally related to compound **31**, previously isolated from leaves of *P. tripartita* and unambiguously elucidated by NMR spectroscopy [19]. The compounds **3** and **8** were structurally related to the diglycoside leucenin-II (compound **5**, MS^n^ in agreement on comparison with a pure standard compound). The compounds **1**, **9**, **14** and **20** relate to standards of vicenin-II (compound **13**) or vitexin (compound **16**). 

**Figure 6:**
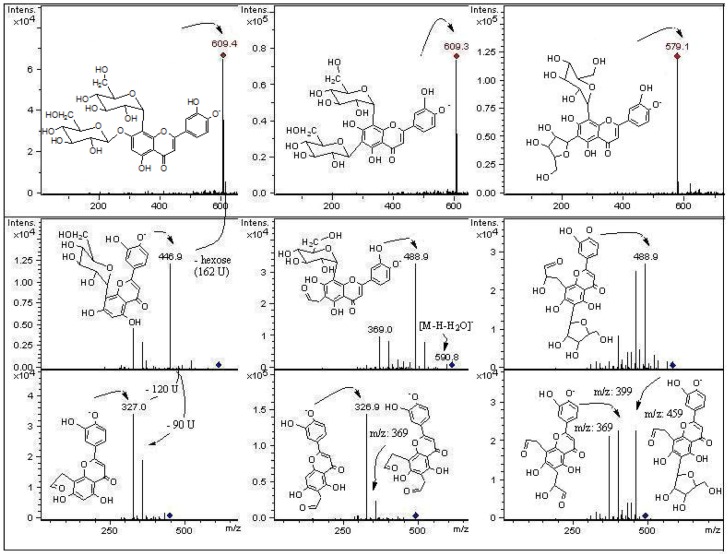
Structures, fragmentation, full ESI-MS and MS-MS spectra of peaks 3, 5 and 8.

**Figure 7:**
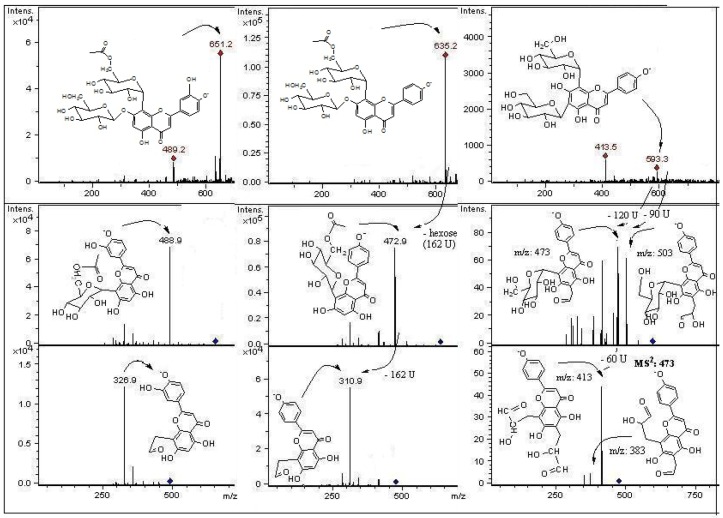
Structures, fragmentation, full ESI-MS and MS-MS spectra of peaks 18, 20, and 13.

The fragmentation behaviour of all detected *C*-glycoside derivatives substituted at positions 6 and 8 were investigated using LC-MS^n^, and the main fragments are shown in Table 2 and Figure 5, Figure 6, Figure 7. The observed fragment ions at *m/z* 179, 151 and 121 evidenced that the aglycone of compounds **3**, **5**, **8**, **18** and **24** was luteolin. The aglycone of compounds **10**, **21** and **31** was luteolin-4′methyl-eter (diosmetin) and fragments at *m/z* 179 and 151 evidenced that the aglycone of compounds **1**, **9**, **13**, **16** and **20** was apigenin. This fact was further corroborated by analyzing pure standards of apigenin, luteolin, orientin, isoorientin, vitexin and isovitexin, which produced the corresponding MS fragments. The proposed identity of all phenolic compounds detected as well as LC-DAD, LC–MS and MS/MS data is depicted in Table 2, and their identification is explained below.

#### 2.2.1. C-Glycosyl Flavones

Peaks **3** and **5** have a full MS spectrum with a [M−H]^−^ ion at *m/z* 609. Peak **5** has a pseudomolecular ion at *m/z* 609 which produced daughter MS ions at *m/z*: 591 (M−H−H_2_O), 489 (M−H−120), MS^2^ at *m/z* 399 (489−90), 369 (489−120 or [M−H−2 × 120]^−^), and 327 (369-42). The retention time, UV spectrum and MS fragmentation agree with that of the di-*C*-hexosyl flavone leucenin-II (luteolin-6,8-di-*C*-*β*-D-glucopyranoside, Figure 3 and Figure 6). The identity was confirmed by spiking experiments with standard compound. The MS spectrum of peak **8** showed mainly fragments at *m/z*: 489, 399, 369, and 327 (Figure 6). The [M−H]^−^ ion at *m/z* 579 in the full MS spectrum of peak **8** pointed out to a pentose instead of an hexose, differing from leucenin-II (peak 5) in one of the *C*-linked sugars. Thus, peak **8** was assigned as a 6-*C*-pentose orientin derivative, tentatively as a luteolin-(6-*C*-pentosyl)-8-*C*-*β*-D-glucopyranoside isomer (Figure 3 and Figure 6). Peak **9** was identified as the apigenin di-*C*-glycoside isoschaftoside (Figure 4) by co-elution with authentic compound. The MS/MS spectrum of peak **10** with a pseudomolecular ion at *m*/*z* 623 showed daughter fragments at *m/z*: 533 ([M−H−90]^−^, 503 [M−H−120]^−^, 413 [M−H−90−120]^−^, and 383 [M−H−2 × 120]^−^), as observed for leucenin II (Figure S5). The fragmentation indicate that compound **10** has the same glycosylation pattern as leucenin II (compound **3**) but with an additional methyl group in the genine, tentatively placed at position 4′. This structural feature was also observed in other methoxylated flavone derivatives (compounds **21** and **31**), tentatively identified as diosmetin derivatives (Figure 3 and Figure 5). Thus compound **10** was identified as leucenin II 4′ methyl ether (or 4′-methoxyluteolin-6,8-di-*C- β*-D-glucopyranoside) as reported previously [27]. Peak **11** was the apigenin di-*C-*glycoside schaftoside with [M−H]^−^ ion at *m/z* 563 U (MS^3^ ions at 473, 442, 383 and 353), while peak **16** was identified as a glucosyl-apigenin (vitexin, Figure S5) with a molecular anion at *m/z* 431, yielding fragments at *m/z* 357, 311 and 283 (Figure 4). In the same manner, peak **13** with a molecular anion at *m/z* 593 and typical fragments at *m/z* 503, 473, 413 and 383 was assigned as apigenin-6,8-di-*C*-*β*-D-glucopyranoside (vicenin II, Figure 7), identity further confirmed by spiking experiments with standard compound. Peak **14** with similar fragmentation pattern was identified as an isomer of the later compound. Peak **17** was identified as eriodyctiol 6,8 di-*C*-glucoside as previously reported [27]. The full MS spectrum of the abundant peak **31** (ion at *m/z *503, [M−H]^−^) was consistent with the molecular formula C_24_H_23_O_12_ and the UV spectrum could be assigned to that of a chrysoeriol, luteolin or diosmetin derivative. Daughter MS ions at *m/z*: 371 (M−H−Ac−90), 341 (M−H−Ac−120) and 299 (M−H−Ac-sugar) (Figure 5) were indicative of the loss of acetyl moiety and cleavages of a glucose moiety of the flavone structure reported for the main compound isolated from the leaves of this plant [19]. The presence of the acetoxyl (at C-6′′) and methoxyl groups (at C-4′) and their location were established on the basis of the NMR spectra reported for this diosmetin derivative and further confirmed with literature data for similar compounds [6]. Peak **21** ([M−H]^−^ ion at *m/z *461) could be assigned as a luteolin glucuronide with a MW of 462. However, it produced similar MS fragments at *m/z *341 (M−H−120), 371 (M−H−90) and 299 (M-H-sugar, diosmetin moiety) as peak **31**, while peak **24** ([M−H]^−^ ion at *m/z *489) showed the deacetylated daughter ion at 447 and prominent *C*-glycoside flavonoid MS^3^ ions at 357 (447-90) and 327 (447-120). Thus, peaks **21** and **24** were assigned as the de-acetyl and de-methyl derivatives of compound **31**: 4′-methoxyluteolin-8-*C*-*β*-D-glucopyranoside and luteolin-8-*C*-(6′′acetyl)-*β*-D-glucopyranoside, respectively (Figure 5). In the same manner, peak **20**([M−H]^−^ ion at *m/z *635, MS^n^ ions at 473 and 311 U) was identified as apigenin-5-*O*-*β*-D-glucopyranosyl-8-*C*-(6”acetyl)-*β*-D-glucopyranoside (Figure 7) while peak **27** with main MS signals at 473 and 311 was identified as apigenin-8-*C*-(6”acetyl)-*β*-D-glucopyranoside.

#### 2.2.2. C- and O-Glycosyl Flavones

The MS/MS fragmentation analysis of peak **1** eluting at Rt = 2.2 min showed a [M−H]^−^ ion at *m/z* 725, while peaks **18** ([M−H]^−^ ion at *m/z* 651) and peak **19** ([M−H]^−^ ion at *m/z* 447, Figure S5) presented MS^n^ fragments indicating loss of 162 U (dehydrated hexose moiety) as reported for *O*-glycosyl flavonoids [28]. Peak **1** with three sugar moieties showed the loss of a dehydrated hexose (162 U) leading to a di-*C*-glucoside assigned as apigenin (6-*C*-*α*-L-arabinopyranosyl)-8-*C*-*β*-D-glucopyranoside (isoschaftoside, this compound peak **9**, was assigned by a spiking experiment with authentic standard). The MS^2^ at *m/z *563 and main MS^3^ ions at *m/z* 443, 383 and 353) support the assignation of compound **1** as the isoschaftoside derivative (6-*C*-α-L-arabinopiranosyl)-7-*O*-glucosyl-8-*C*-*β*-D-glucopyranoside (Figure 4). Peak **3** with a molecular anion at *m/z* 609 showed a MS^2^ ion at *m/z *447 (orientin moiety) after the loss of a dehydrated hexose (M−H−162), tentatively attached at position 7 of the orientin structure. The loss of the hexose as a dehydrated sugar residue (162 U) rather than an entire sugar molecule (180 U for hexose) for this compound suggested that this additional sugar moiety could be linked at one of the phenolic OH groups of the flavonoid structure instead to an OH of the primary sugar, as previously reported for flavonols bearing a branched glycosyl group with glucose or galactose as the primary sugar [29,30]. The fragment at *m/z* 447 showed the characteristic orientin MS^3^ ions at *m/z* 357 (M−H−90), and 327 (M−H−120). The compound was tentatively assigned as luteolin-(7-*O*-glucopyranosil)-8-*C*-glucopyranoside (orientin-7-*O*-glucoside, Figure 6). Peak **23** with UV data compatible with a flavonol glycoside, pseudomolecular ion at *m*/*z *631 and MS-MS ions at *m/z* 479 and 317 was identified as myricetin-3-*O*-(6” galloyl) galactoside as reported [31]. Peak **25** with a pseudomolecular ion at *m*/*z*691 and fragment at *m*/*z *631 (myricetin-3-*O*-(6” galloyl) galactoside, and 479 (myricetin-3-*O* galactoside) was identified as a myricetin-3-*O*-(6” galloyl) glycoside derivative.

**Table 2:** Identification of phenolic compounds in tumbo fruits by LC-DAD, LC–MS and MS/MS data.

Peak	Rt(min)	λ max (nm)	[M−H]^−^	Fragment ions (*m/z*)	Compound identification	Fruit part
1	2.1	269, 340	725	665, 563, 443, 383, 353	(6-*C*-α-L-arabinopiranosyl)-7-*O*-glucosyl-8-*C*-*β*-D-glucopyranoside (7-*O*-glucosyl-isoschaftoside)	P, J
2	2.8	288, 322	897	457, 451, 325, 305	Feruloylated oligosaccharide	P, J
3	9.4	270, 349	609	489, 369, 327	Luteolin-(7-*O*-glucopyranosil)-8-*C*-glucopyranoside (Orientin-7-*O*-glucoside	P
4	9.6	270, 349	645	447, 357, 327	Luteolin-di-glycoside derivative	P
5	9.9	269, 350	609	447, 357, 327	Luteolin-6,8-di-*C*-*β*-D-glucopyranoside (Leucenin II) *	P
6	10.2	269, 335	629	593	Vicenin II derivative	P
7	10.7	-	533	371	5′-Methoxy-demethylpiperitol-4-*O*-glucoside	P, J
8	14.0	269, 349	579	489, 459, 399, 369	Luteolin-(6-*C*-pentosyl)-8-*C*-*β*-D-glucopyranoside isomer	P, J
9	14.1	269, 337	563	503, 473, 443, 383, 353	(6-*C*-α-L-arabinopiranosyl)-8-*C*-*β*-D-glucopyranoside (Isoschaftoside) *	P
10	14.3	271, 346	623	533, 503, 413, 383	4′-Methoxyluteolin -6,8-di-*C-β*-D-glucopyranoside(Leucenin II, 4′-methyl ether)	P
11	14.6	269, 337	563	473, 353	Apigenin (6-*C*-*β*-D-glucopyranosyl) 8-*C*-α-L-arabinoside (Schaftoside) *	P, J
12	14.7	270, 348	687	651,489, 327	Luteolin-5-*O*-glucosyl-8-*C*-(6”acetyl)-*β*-D-glucopyranoside derivative	P
13	15.6	269, 335	593	503, 473, 413, 383	Apigenin-6,8-di-*C*-*β*-D-glucopyranoside (Vicenin II) *	P, J
14	16.2	269, 335	593	503, 473, 413, 383	Vicenin II isomer	P, J
15	16.8	270, 340	523	361	Unknown di- glucosyl flavonoid	P, J
16	17.5	269, 338	431	357, 311, 283	Apigenin-8-*C*-*β*-D-glucopyranoside (Vitexin) *	P
17	18.0	-	611	593, 491, 429, 393, 369, 327	Eriodictyol 6,8 di-*C*-glucoside	P
18	18.3	270, 348	651	489, 327	Luteolin-7-*O* -glucopyranosyl 8-*C*-(6”acetyl)-glucopyranoside	P, J
19	18.6	270, 349	447	285	Luteolin-8-*C*-*β*-D-glucopyranoside (Orientin) *	P,J
20	17.0	269, 338	635	473, 311	Apigenin-5-*O*-*β*-D-glucopyranosyl, 8-*C*-(6”acetyl)-*β*-D-glucopyrano- side	P, J
21	20.2	270, 347	461	285, 216	4′-Methoxyluteolin-8-*C*-*β*-D-glucopyranoside	P, J
22	20.4	257, 361	317	300, 179, 151	Myricetin *	P
23	21.3	257, 361	631	479, 317	Myricetin-3-*O*- (6”-galloyl) glycoside	P
24	24.5	270, 350	489	447, 327	Luteolin-8-*C*-(6”acetyl)-*β*-D-glucopyranoside	P, J
25	24.7	257, 361	691	631, 479, 335, 317, 273	Myricetin-3-*O*-(6” galloyl) glycoside derivative	P
26	25.1	257, 361	331	315, 300, 179, 151	Myricetin 3′ methyl ether	P
27	25.7	269, 338	473	413, 311	Apigenin-8-*C*-(6”acetyl)-*β*-D-glucopyranoside	P
28	27.6	270, 335	713	677, 533, 451, 337	Unknown *C*-glycosyl derivative	P
29	28.4	270, 335	723	677, 533, 451, 337	Unknown *C*-glycosyl derivative	P, J
30	28.7	272, 330	857	501, 337	Unknown *C*-glycosyl derivative	P
31	29.1	269, 346	503	371, 341, 299	4′- Methoxyluteolin-8-*C*-(6”acetyl)-*β*-D-glucopyranoside *	P, J

Fruit part: P: peel, J: pulp and juice. * Identified by spiking experiments with a standard compound.

#### 2.2.3. Other Phenolic Compounds

Peak **2** with a pseudomolecular ion at 897 U and UV maxima at 322 nm, compatible with ferulic acid, produced in the ion trap MS daughter ions at *m/z*: 457, 451, 325, 305 and was assigned as a feruloyl-oligosaccharide composed by four arabinose residues associated with two ferulic acid moieties, as reported from sugar beet (*Beta vulgaris*) [32]. Peak **7** with a pseudomolecular ion at *m/z* 533 which yielded a main MS^2^ ion at *m/z* 371 was tentatively identified as 5′-methoxy- demethylpiperitol-4-*O*-glucoside as reported [33]. 

#### 2.2.4. Unidentified Compounds

Peaks **4**, **12**, **15**, **28**, **29** and **30** remain unidentified. Peak **4** showed a similar UV spectra to peak **3** (λ_max_ 270, 349). The mass spectrum of peak **4** with a [M−H]^−^ ion at *m/z* 645 yielded MS ions at *m/z* 609, 447, 357 and 327 and was tentatively characterized as a luteolin-di-glycoside derivative with an unidentified additional sugar moiety. Peak **12** with a [M−H]^−^ ion at *m/z* 687 produced ions at *m/z* 651, 489, 327 which matches the MS data found for peak **18** (luteolin-7-*O*-glucopyranosyl, 8-*C*-(6′′acetyl)-glucopyranoside, full MS at *m/z* 651) after the loss of 38 amu and thus was tentatively assigned as an unknown derivative of compound **18**. Peak **15** with a ion at *m/z* 561 which produced an MS^2^ ion at *m/z* 361 was also reported to occur in *Ginkgo biloba* extract [34] and was assigned to an unidentified glycosyl flavonoid derivative (λ_max_ 270, 340). Peaks **28**, **29** and **30 **showed pseudomolecular ions of *m/z* 713, 723 and 857 U respectively. A flavanone derivative with pseudomolecular ion at *m/z* 723 was identified in bergamot juice as naringin di-oxalate [35]. However, compound **29** ([M−H]^−^ ion at *m/z* 723) as well as **28** and **30** showed UV spectra compatible with a bi- *C*-glycosyl-flavone (Table 1). We were not able to match a flavonoid structure for the full mass and fragmentation patterns (MS^n^ at 677, 533, 451 and 337) observed for compounds **28** and **29** as well as for 501 and 337 found for compound **30**. 

### 2.3. Total Phenolic, Total Flavonoid Content and Antioxidant Power of Tumbo Fruits

The peel of tumbo fruits cultivated in the Oasis de Pica, Chile, showed total phenolic content of 56.03 ± 4.34 mg per 100 g dry material (Table 1). This value is 2.5 times higher than the content in pulp and juice (22.57 ± 1.43 mg/ 100 g dry material). However, the value was lower to that reported for a sample from Perú (2.16 mg GAE/100 g fresh weight) [16]. The total flavonoid content of the peel was 1.81 times higher than that of the pulp and juice (140.17 ± 4.2 *versus* 77.16 ± 8.4 mg per 100 g dry weight, respectively). The antioxidant power measured as the DPPH scavenging capacity and the ferric reducing antioxidant power (FRAP) was higher in the peel with values of 10.41 µg/mL and 462.47 mmol/100 g dry weight than in the pulp (12.89 µg/mL and 85.78 mmol/100 g dry weight, respectively) (Table 2). The values correlates well with the total phenolic, total flavonoid as well as with the number and intensity of phenolics detected in the extracts (Figure 3, Table 1). When comparing with fruits from other South American countries, the FRAP capacity of the pulp and peel were lower than that reported for a tumbo sample from Colombia (114 ± 3.28 and 42.2 ± 2.29 µmol TE/g fresh weight, respectively) [36]. The known *C*-glycosyl flavonoids isolated shower high antioxidant power (Table 2) as previously reported [37] while the acetylmethyl orientin derivative **31** showed two times less DPPH bleaching capacity than its related compound orientin (**19**), probably due to the substitution of an OH group by a OCH_3_ function.

## 3. Experimental

### 3.1. General

HPLC grade methanol, ethyl acetate and 1-butanol were purchased either from Merck (Darmstadt, Germany) or from J.T. Baker (Phillipsburg, NJ, USA). Aluminum-coated silica gel thin layer chromatography (TLC, Kieselgel F_254_) plates and formic acid from Merck. Sephadex LH-20 was obtained from Pharmacia Fine Chemicals (Piscataway, NJ, USA). Amberlite XAD-7HP 20-60 mesh resin, quercetin, 1,1-diphenyl-2-picrylhydrazyl (DPPH^.^), and gallic acid were purchased from Sigma Chemical Co. (St. Louis, MO, USA). Deuterated MeOH, HPLC water, HCl, KCl, Folin–Ciocalteu phenol reagent, sodium acetate, aluminum chloride hexahydrate and sodium carbonate were from Merck. Vicenin II, leucenin II, vitexin, orientin, isovitexin, homoorientin, schaftoside, myricetin and isoschaftoside for HPLC analysis all with purity higher than 95% (with HPLC certificate) were purchased either from ChromaDex (Santa Ana, CA, USA) or Extrasynthèse (Genay, France).

LC-DAD analyses were carried out using a Merck-Hitachi equipment with a quaternary L-7100 pump, a L-7455 UV diode array detector, and a D-7000 chromato-integrator (LaChrom, Tokyo, Japan). A 250 × 4.6 mm i.d., 5 *μ*m, Purospher star-C18 column (Merck) set at 25 °C was used for the separation of all phenolics. Detection was carried out at 280 and 365 nm, with peak scanning between 200 and 600 nm. Gradient elution was performed with water/1% formic acid (solvent A) and acetonitrile/1% formic acid (solvent B) at a constant flow rate of 1.0 mL/min. An increasing linear gradient (v/v) of solvent B was used [*t* (min), % A]: 0, 90; 4, 90; 25, 75; 40, 90. For LC-ESI-MS analysis an Esquire 4000 Ion Trap mass spectrometer (Bruker Daltoniks, Bremen, Germany) was connected to an Agilent 1100 HPLC (Agilent Technologies, Waldbronn, Germany) instrument via ESI interface. A Bruker Daltoniks 3.2 data analysis software was used for acquisition and processing. Full scan mass spectra were measured between *m/z* 150 and 2000 U in negative ion (preferred) mode. Nitrogen was used as nebulizer gas at 27.5 psi, 350 °C and at a flow rate of 8 L/min. The mass spectrometric conditions were: electrospray needle, 4000 V; end plate offset, −500 V; skimmer 1, −56.0 V; skimmer 2, −6.0 V; capillary exit offset, −84.6 V. Collision induced dissociation (CID) spectra were obtained with a fragmentation amplitude of 1.00 V (MS/MS) using ultrahigh pure helium as the collision gas.

Preparative HSCCC was carried out using a Quattro AECS-QuikPrep™ coil planet centrifuge high-speed counter-current chromatograph model MK-7 (AECS- QuikPrep Ltd., Bristol, UK) equipped with four stained steel coils of 2.1 mm I.D. and 25, 210, 115 and 115 mL capacity, respectively. All coils could be used individually, or as any combination of numbers in series or in parallel. We used the 115 mL preparative coil whose *β* values range from 0.62 at internal to 0.82 at the external .The mobile phase was pumped using two series II HPLC pump (Scientific Systems, Inc., State College, PA, USA). The sample was injected using a 7 mL loop through a rheodine valve, and solvents for the gradient run were switched through a switching valve. An UV detector (ECOM-Flash 06 S single wavelength, Prague, Czech Republic) at 254 nm was used after the HSCCC machine, and chromatograms were visualized using ECOMAC software. The proton and carbon (^1^H: 400 MHz; ^13^C: 100.25 MHz) NMR experiments were performed in a Bruker Avance 400 UltraShield spectrometer using CD_3_OD or deuterated DMSO as solvent. The spectroscopic measurements were performed using a Unico 2800 UV-vis spectrophotometer (Unico Instruments, Co, Ltd., Shangai, China). 

### 3.2. Plant Material

The study was undertaken with ripe banana passion (*P. tripartita* var. mollissima, local name tumbo) fruits cultivated at the Oasis de Pica (I Region of Chile) in January 2011. The ripe fruits were immediately processed upon arrival to the laboratory. A lyophilized sample is deposited at the Laboratorio de Productos Naturales, Universidad de Antofagasta, under reference number Pt15-01-12.

#### 3.2.1. Extraction of Tumbo Fruits

Fresh ripe fruits (1.98 kg, Figure S6) were carefully washed. The peel, pulp-juice and seeds were manually separated to yield 1,127 g of pulp and juice and 555 g of peel. The pulp/juice and peel were lyophilized yielding 110.44 g (9.80% w/w) and 61.0 g (10.99% w/w) of dry material, respectively. The lyophilized pulp-juice and peel were homogenized in a mortar and extracted three times with MeOH/H_2_O 7:3 v/v (3 × 1,000 mL and 3 × 500 mL each, respectively) at room temperature in the dark using an ultrasound bath (25 °C, 40 Hz), for 1 h per extraction. Extracts were combined, filtered and concentrated under reduced pressure below 50 °C. Afterwards the extracts were suspended in water, filtered using a Buchner funnel and separately loaded onto an Amberlite XAD7 HP (500 and 300 g, respectively) resin column. This polymeric resin was used for pre-concentration of ionic and small polar compounds. Amberlite XAD7 HP can adsorb all phenolic compounds which can be desorbed with a polar organic solvent. The column was rinsed with 2 L and 1 L respectively of HPLC grade water and compounds were desorbed-eluted with 1 L MeOH for each extract. Both eluates were concentrated under reduced pressure below 45 °C to give 4.5 g (0.22% w/w yield) of a brown extract for the pulp-juice and 3.2 g (0.16% w/w yield) of a yellow-brown extract for the peel. 

#### 3.2.2. Isolation and Characterization of Phenolics from the Peel Extract

The peel extract obtained as explained above was subjected to gradient preparative high speed centrifugal countercurrent chromatography (HSCCC). First, a suitable solvent system was chosen by HPLC and TLC according to the partition coefficients (0.5 < K < 2.0) between a series of biphasic solvent systems. The solvent systems were mixtures of *n*-hexane–ethyl acetate–methanol–water, ethyl acetate–*n*-butanol–water and ethyl acetate–*n*-butanol–water-acetic acid using different (v:v:v:v), ratios as follows. About 2.0 mg of tumbo peel extract was added to each test tube, and then 2 mL of each phase of a pre-equilibrated two-phase solvent system was added and thoroughly mixed. Each test tube was shaken with a vortex mixer for one minute and left to stand at room temperature until equilibrium was attained between the two phases. Then, the upper and lower phases were analyzed by TLC and HPLC at 280 nm to obtain K values of all flavonoid target compounds. The partition coefficient (K) is defined as K = AUp/ALo, where AUp and ALo were the HPLC peak areas of glycoside compounds or TLC spot areas from the upper and lower phase, respectively. The solvent system: ethyl acetate–n-butanol–water in a 1:1:1 v/v/v ratio provided the better K values for all mayor compounds (0.9 < K < 1.3). In order to perform gradient preparative HSCCC, four two-phase solvent systems composed of ethyl acetate (EtOAc), *n*-butanol (BuOH), and water (W) were prepared as follows. Solvent A: EtOAc–BuOH–W 1:1:1 v/v/v ratio, solvent B: EtOAc–BuOH–W 1:0.5:1 v/v/v ratio, solvent C: EtOAc–BuOH–W 1:0.3:1 v/v/v ratio and solvent D: EtOAc–BuOH–W 1:0.2:1 v/v/v ratio, respectively. The solvent systems were prepared, shaken in a funnel, equilibrated overnight, and the two phases separated shortly before use. The first lower aqueous phase (solvent system A) served as the stationary phase and was pumped into the coil until it was filled with no rotation. The first mobile phase (A) was then pumped in a tail to head mode with the planetary rotor at 850 rpm at a flow rate of 2.0 mL/min and 25 °C. When only mobile phase emerged from the column, the percentage retention of the stationary phase was recorded (62%) and then 1.0 g of filtered Tumbo peel extract (dissolved in 3.5 mL of mobile and 3.5 mL of stationary phase) was injected into the coil through an injection loop using a Rheodine valve. The mobile upper phase was switched from A to D using a switching valve every 60 minutes. The effluent from the outlet of the column was continuously monitored with a UV detector at 254 nm (see experimental) and four (T1-T4) fraction pools were collected (Figure 2). After stopping rotation, the stationary phase contained an unresolved complex mixture of polar flavonoids as verified by NMR spectroscopy. The four flavonoid-enriched fractions were concentrated under reduced pressure and then purified by permeation on Sephadex LH-20 columns (10% loading capacity) using HPLC grade MeOH as eluent. The fractions collected from the open column permeation were compared by analytical TLC using BuOH-AcOH-H_2_O in a 80:25:4 ratio as the mobile phase. The chromatograms were visualized under UV light (254 nm) and then sprayed with 1% vanillin in EtOH (w/v) and heated (60 °C) to see the compound spots. From fractions T1 (35 mg), T2 (42 mg), T3 (135 mg) and T4 (37 mg), the following pure compounds were obtained: **11** (schaftoside, 12 mg), **13** (vicenin II, 14 mg), **16** (vitexin, 7 mg), **19** (orientin, 24 mg), and **31** (4′-methoxy-luteolin-8-*C*-(6′′acetyl)- *β*-D-glucopyranoside, 27 mg).

### 3.3. Polyphenolic Content

A precisely weighed amount of each extract (approximately 1 mg/mL) as explained in Section 3.3 was used for total phenolic (TPC) and total flavonoid (TFC) content. Extracts were dissolved in a MeOH-water 7:3 v/v solution. Appropriate dilutions were prepared and absorbance was measured using a spectrophotometer (see section 3.1). The TPCs were determined by the Folin and Ciocalteu’s reagent method [38]. Briefly, the appropriate extract dilution was oxidized with the Folin-Ciocalteu reagent (2 mL, 10% v/v), and the reaction was neutralized with sodium carbonate. The calibration curve was performed with gallic acid (concentrations ranging from 16.0 to 500.0 μg/mL, R2 = 0.999). The absorbance of the resulting blue color of the complex formed was measured at 740 nm after 30 min., and the results were expressed as mg of gallic acid equivalents per 100 g dry material. The TFCs in the samples were determined as previously reported [39]. The absorbance of the reaction mixture (2.5 mL) was measured at 430 nm and quercetin was used as a reference for the calibration curve (concentrations ranging from 16.0 to 800.0 µg/mL, R2 = 0.994). Results were expressed as mg quercetin equivalents per 100 g dry weight. Data are reported as mean ± SD for at least three replicates. 

### 3.4. Antioxidant Assessment

#### 3.4.1. Bleaching of the 2,2-Diphenyl-1-picrylhydrazyl (DPPH) Radical Assay

Free radical scavenging capacity was evaluated according to the method described previously. Aliquots of samples (100 μL) were assessed by their reactivity with a methanol solution of 100 μM DPPH. The reaction mixtures (2 mL) were kept for 30 min at room temperature in the dark. The decrease in the absorbance (n = 3) was measured at 517 nm, in a Unico 2800 UV-vis spectrophotometer (Unico Instruments, Co, Ltd, Shanghai, China). The percent DPPH scavenging ability was calculated as: DPPH scavenging ability = (A_control_ – A _sample_/A_control_) × 100. Afterwards, a curve of % DPPH scavenging capacity *versus* concentration was plotted and IC_50_ values were calculated. IC_50_ denotes the concentration of sample required to scavenge 50% of DPPH free radicals. The lower the IC_50_ value the more powerful the antioxidant capacity. If IC_50_ ≤ 50 μg/mL the sample has high antioxidant capacity, if 50 μg/mL < IC_50_ ≤ 100 μg/mL the sample has moderate antioxidant capacity and if IC_50_ > 200 μg/mL the sample has no relevant antioxidant capacity. In this assay, the standard antioxidant compound gallic acid showed an IC_50_ value of 1.16 μg/mL (6.81 μM). 

#### 3.4.2. Ferric Reducing Antioxidant Power (FRAP) Assay

The FRAP assay was done according to [40] with some modifications. The stock solutions included 300 mM acetate buffer pH 3.6, 10 mM TPTZ (2,4,6-tripyridyl-*s*-triazine) solution in 40 mM HCl, and 20 mM FeCl_3_·6H_2_O solution. The working solution was prepared by mixing 50 mL acetate buffer, 10 mL TPTZ solution, and 15 mL FeCl_3_.6 H_2_O solution and then warmed at 37 °C before using. Tumbo fruit extracts (100 μL) were allowed to react with 2 mL of the fresh FRAP solution for 30 min in the dark. Readings of the coloured product ferrous tripyridyltriazine complex were then taken at 593 nm (n = 3). The standard curve was performed with the standard antioxidant Trolox (R² = 0.9995). Results are expressed in mM TE (Trolox equivalents)/100 g dry mass. 

### 3.5. Statistical Analysis

The statistical analysis was carried out using the originPro 9.0 software packages (Originlab Corporation, Northampton, MA, USA). The determination was repeated at least three times for each sample solution. Analysis of variance was performed using ANOVA. Significant differences between means were determined by student’s t-test (*p* values < 0.05 were regarded as significant).

## 4. Conclusions

The juice and pulp of banana passion fruit (*P. tripartita*), locally known as “tumbo” and cultivated in northern Chile, were investigated for phenolic and flavonoid content, antioxidant capacity and phenolic compounds composition using hyphenated techniques. The fruit peel showed higher antioxidant power and higher content of phenolics than the pulp and juice. Thirty one phenolic compounds were detected in tumbo peel by HPLC-DAD and ESI-MS/MS analysis. From them, eighteen compounds were characterized as *C*-glycosyl flavonoids and four were *O*-glycosyl flavonoids. Only fifteen out of the 31 phenolics found in the peel were detected in the juice and pulp, namely compounds **1**, **2**, **7**, **8**, **11**, **13**–**15**, **18**–**21**, **24**, **29** and **31**. Compounds **1**, **3**, **5**, **8**–**11**, **13**, **16**, **18**–**21**, **27** and **31** were assigned as *C*-glycosyl flavonoids, while compounds **10**, **21**, **31** are methoxyluteolin derivatives. Five compounds (**11**, **13**, **16**, **19** and **31**) were isolated from the fruit peel extract by HSCCC and identified by spectroscopic and spectrometric means. This method proved to be suitable for the preparative isolation of polar constituents of tumbo fruits and can be applied to other mixtures of close related *C*-glycosyl flavonoids. Compounds **18** (main compound) and **20** are acetyl *C*-glycosyl flavonoids and are reported for the first time in the genus *Passiflora*. 

The whole fruit, including peel, pulp and juice, presented high antioxidant power, which is related to the high total phenolic and flavonoid content and number of healthy *C*-glycosides detected and characterized in the samples. The polyphenolic fingerprint described in this report and the occurrence of unusual chemical markers such as compounds **10**, **21** and **31** can be used to differentiate *Passiflora tripartita* var. *mollissima* from other *Passiflora* species containing flavonoid *C*-glycosides. Most of the compounds described in this article are reported for the first time in *P. tripartita* except peak 31. This study presents the first comprehensive examination of phenolics in banana passion fruits and encourages further studies on the nutraceutical properties of Andean edible fruits as new crops with beneficial effects in the health of consumers worldwide.

## Supplementary Materials

Supplementary materials can be accessed at: http://www.mdpi.com/1420-3049/18/2/1672/s1.
