# Supplementary material for: The Passiflora tripartita (Banana Passion) Fruit: A Source of Bioactive Flavonoid C-Glycosides Isolated by HSCCC and Characterized by HPLC–DAD–ESI/MS/MS
Source: Molecules. 2013 Jan 28;18(2):1672–92. doi: 10.3390/molecules18021672 (PMC6270644; doi:10.3390/molecules18021672)

Supplementary Materials

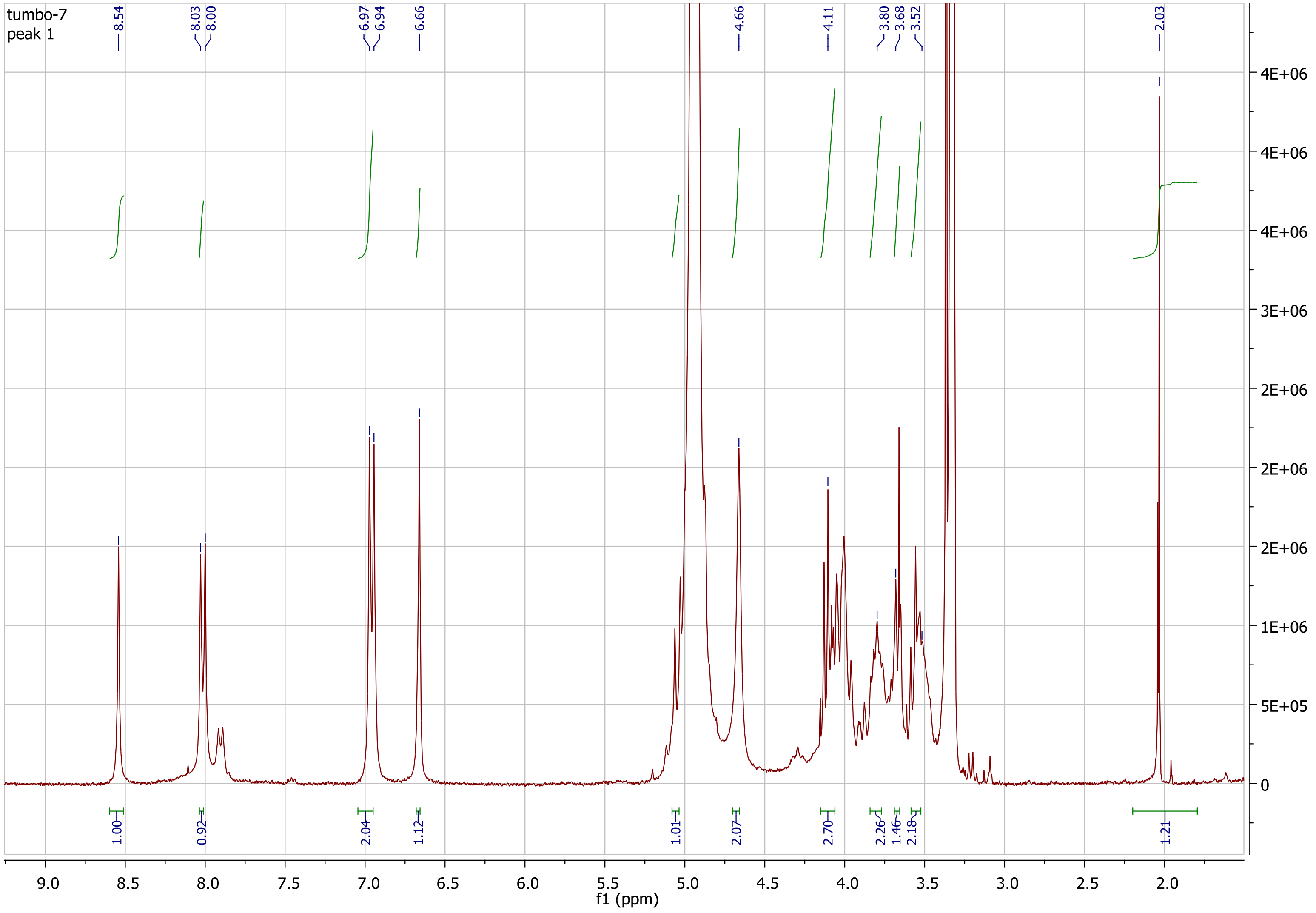

Figure S1. Proton NMR spectra of compound **31**. (CD_3OD and $(CD_3)_2SO$, 400 MHz).

Figure S2. Proton NMR spectra of compound **31**. (CD_3OD, 400 MHz).

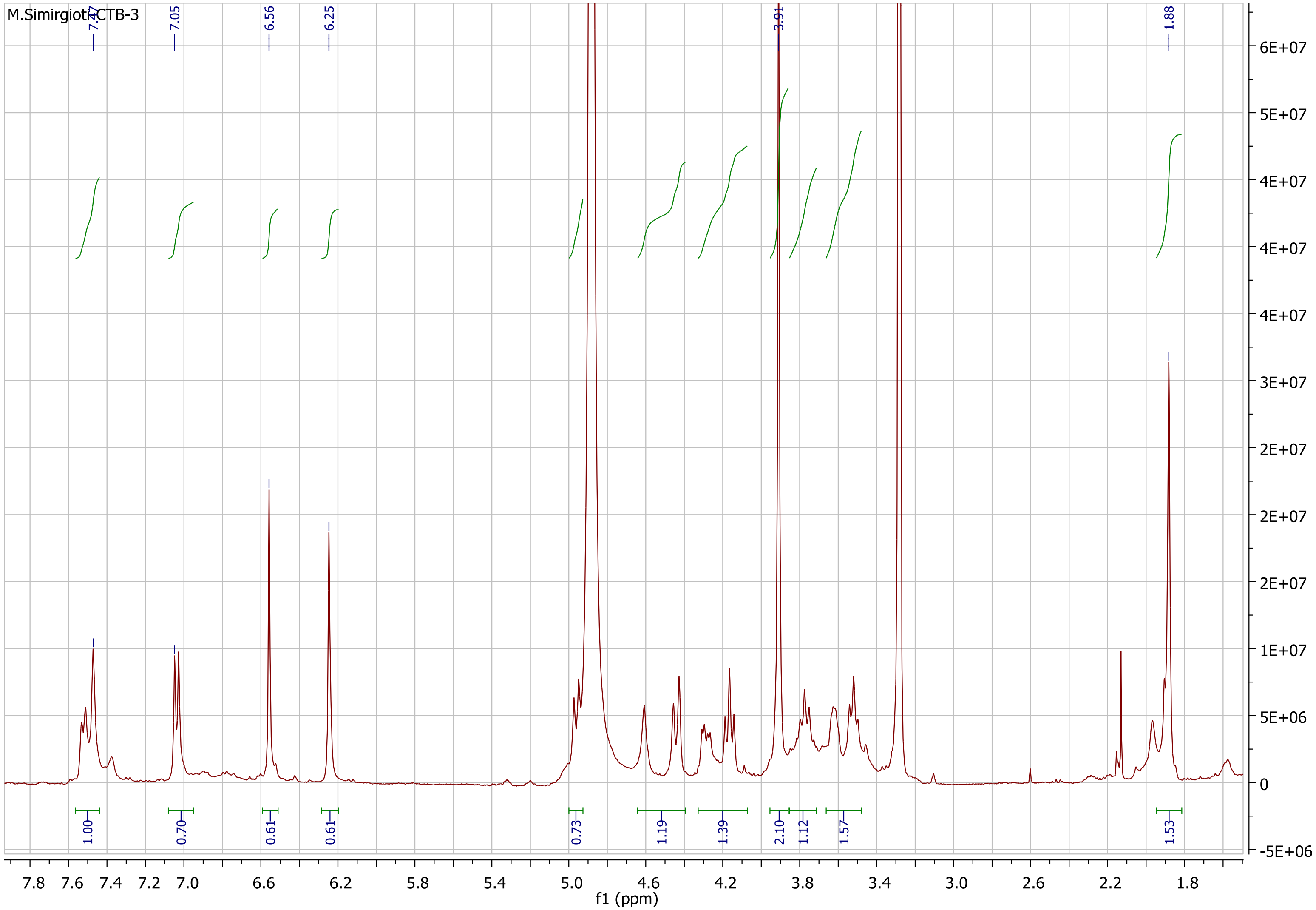

Figure S3. H-H COSY NMR spectra of compound **31**. (CD_3OD, 400 MHz).

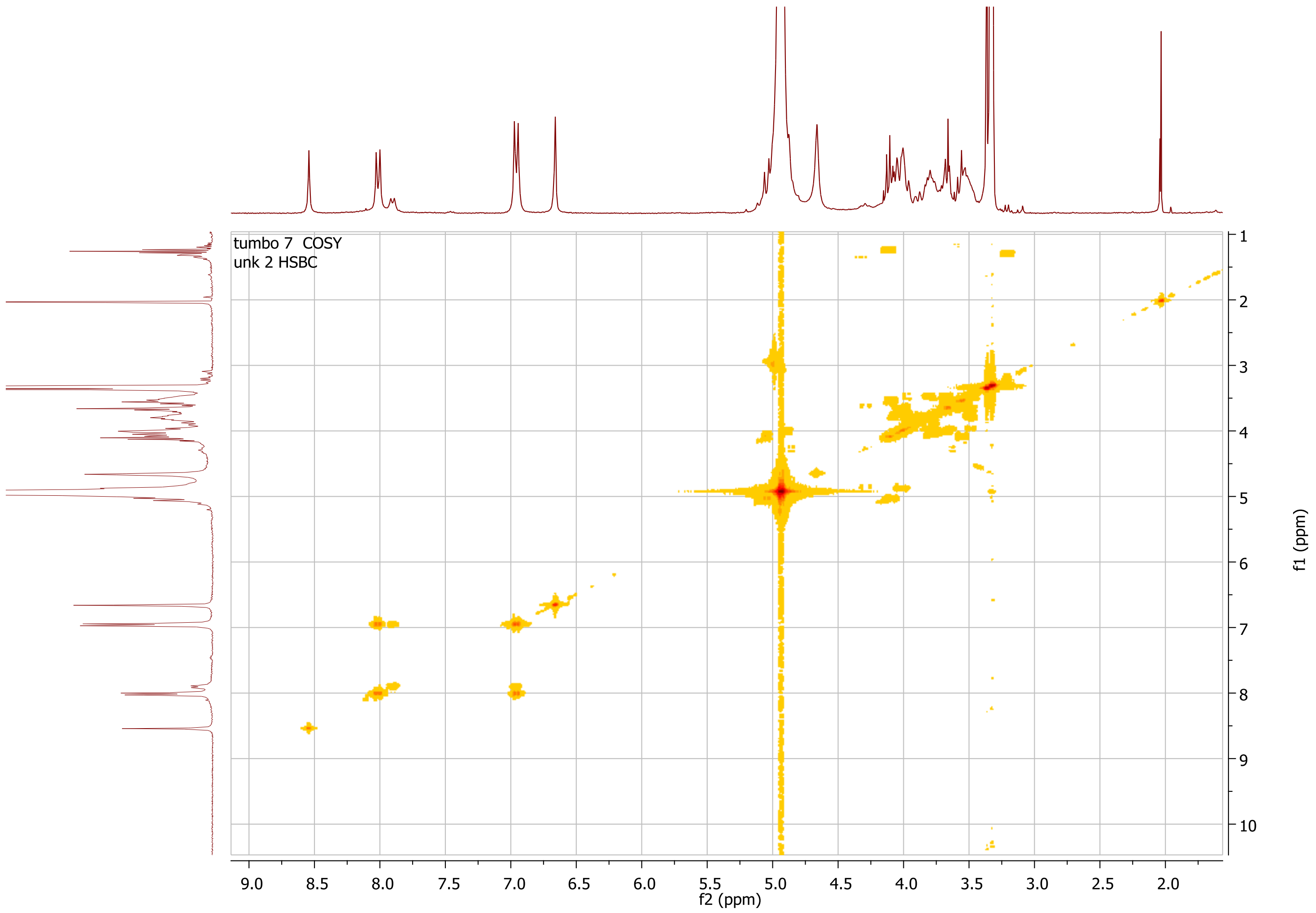

Figure S4. Ampliation in the sugar region of ^{1}H-^{1}H COSY NMR spectra of compound **31**. (CD_3OD, 400 MHz).

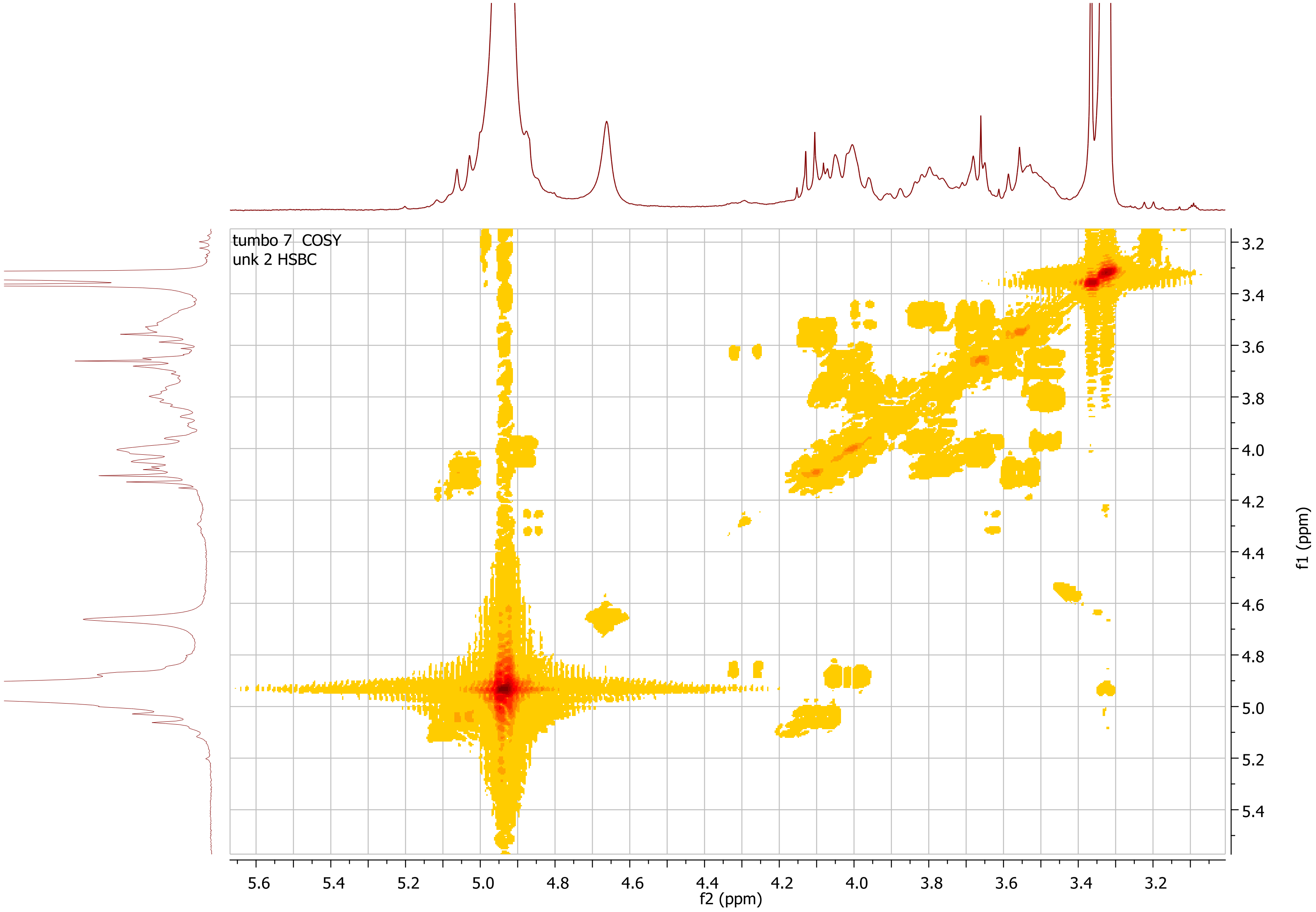

Figure S5. Full ESI MS spectra and MS^n fragmentation of Peaks **10** (Leucenin II, 4¨methyl-eter), **16** (vitexin), and **19** (orientin) from Tumbo peel extracts.

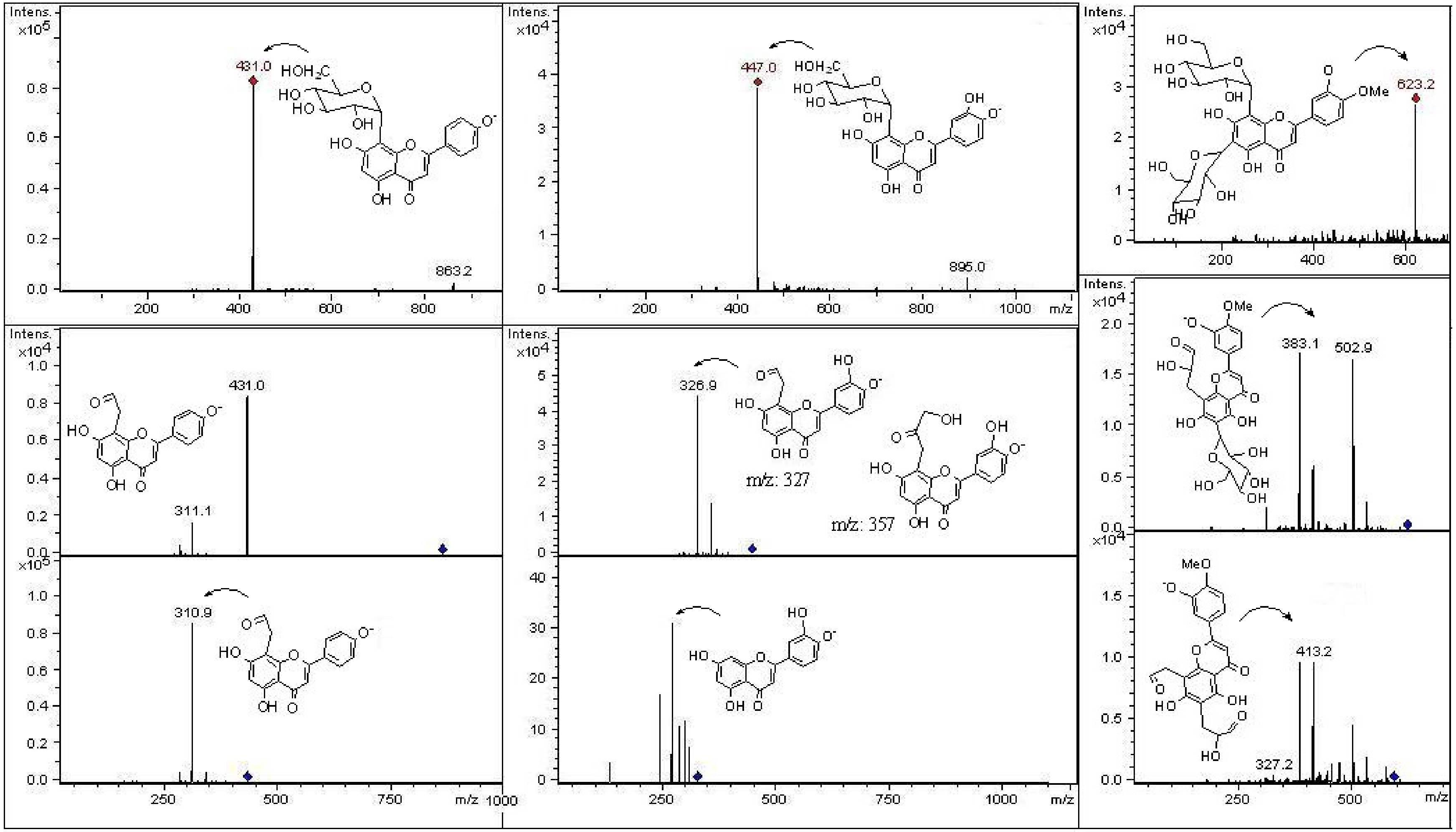

Figure S6. Photograph of ripe tumbo fruits collected in Pica, Chile in 2011.

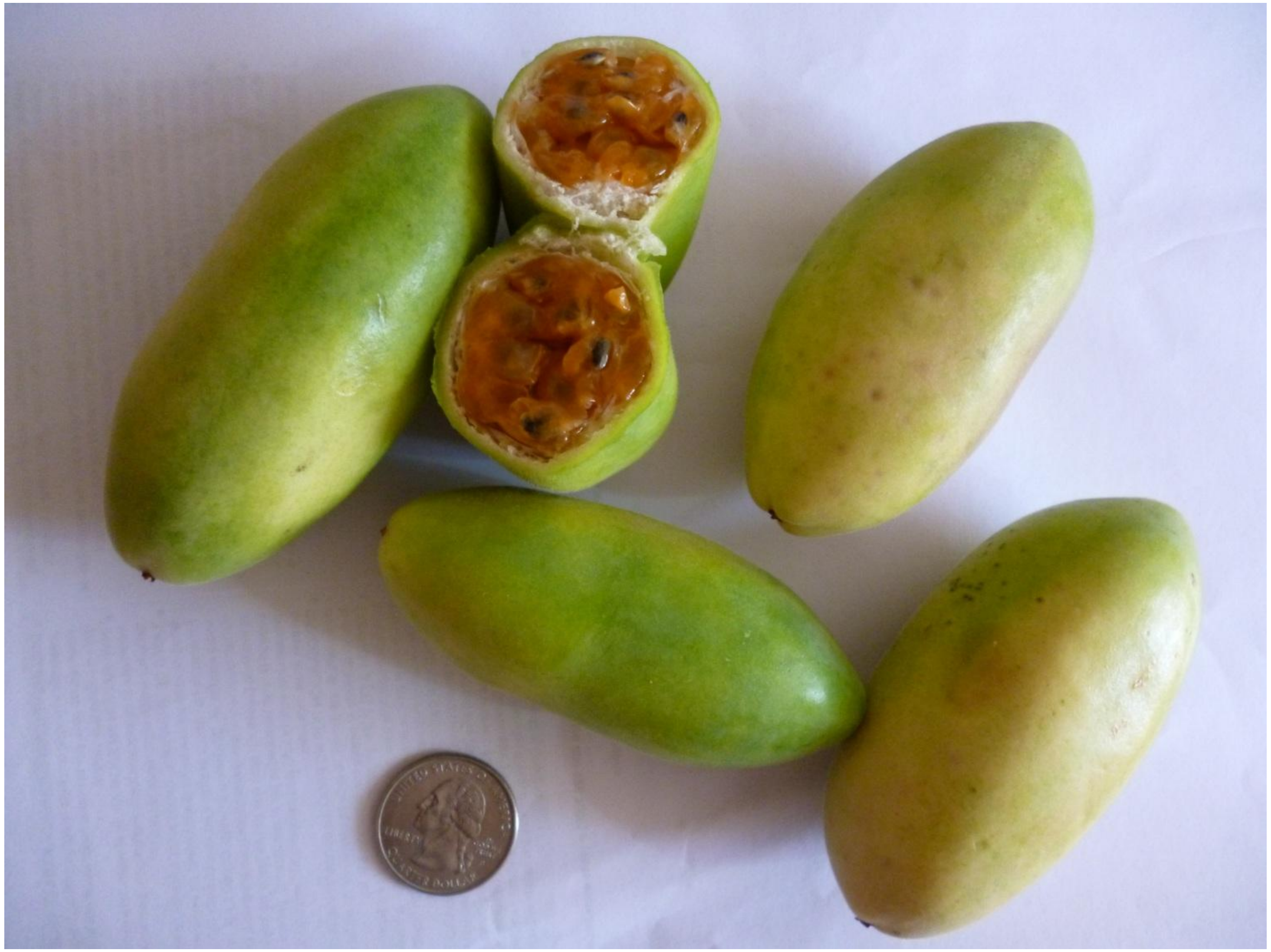

Supplement: Supplementary file 1 [file molecules-18-01672-s001.pdf]
